# The Behaviour of Stratified Fabrics of Aramid Fibres under Stabbing Conditions

**DOI:** 10.3390/polym16070882

**Published:** 2024-03-23

**Authors:** Lorena Deleanu, Viorel Totolici Rusu, George Ghiocel Ojoc, George Catalin Cristea, Mihail Boțan, Alexandru Viorel Vasiliu, Christian Popescu

**Affiliations:** 1 Department of Mechanical Engineering, Faculty of Engineering, “Dunarea de Jos” University, 800201 Galati, Romania; viorel.totolici@ugal.ro (V.T.R.); alexandru.vasiliu@ugal.ro (A.V.V.); 2 National Institute for Aero-Space Research (INCAS) “Elie Carafoli”, 061126 Bucharest, Romania; cristea.george@incas.ro (G.C.C.); botan.mihail@incas.ro (M.B.); 3 Center for Research and Innovation for CBRN Defense and Ecology (CRICBRNDE), 041309 Bucharest, Romania; chr_popescu@yahoo.com

**Keywords:** aramid fabric, SRM509, stab, penetration height, maximum force, time-of-stab impact

## Abstract

This paper presents research on several factors influencing the stabbing behaviour of stratified panels made of aramid fabric Twaron^®^ SRM509 Teijin Aramid BV (Arnhem, The Netherlands). The inputs in the test campaign were the number of layers, the impact energy, and the sample size. Tests were performed on small samples (130 mm × 130 mm) on an Instron^®^ CEAST 9350 drop-tower impact system (Norwood, MA, USA) and on larger samples (400 mm × 400 mm) using a test installation with the same values of the impact energy. Knife type S1 was used, with the geometry recommended in NIJ Standard 0115.00 Stab Resistance of Body Armor SEM, and macro photography investigations revealed the failure mechanisms of panel, layers and fibres. A very important conclusion of this study regarding the stabbing performance of fabric Twaron^®^ SRM 509 in particular, but also in general for panels for body protection is that a research study could start on small size samples, with an accurately instrumented machine, in order to establish the influence of significant factors of stab resistance (energy level, number of layers in a panel, etc.), as these samples are less expensive and less time consuming, but the study should be continued to examine larger size samples. The obtained data are useful for the prototype.

## 1. Introduction

Knives of different shapes and materials have been used since prehistoric times as weapons in combat, tools in various processes, such as construction, and utensils for survival and food preparation. They have become a basic tool that allowed for the development technology, culture, science, and the military.

Body armour has two main goals: to maximise both the survivability of the wearer and their mobility on the battlefield. These could be achieved by maximising the energy absorption and dissipation, maximising the freedom of movement and minimising deformation and penetration (thus protecting the body from being injured) [1,2].

Proposed solutions for stab protection include panels inspired by animal protection [3,4,5,6,7,8,9], either in the form of whole boards [10], profiled, or panels made of scale-like components [11,12,13], eggshells [14], or pads [15,16,17], their production being possible even by additive manufacturing [18,19,20]. Research has also been reported on vests with fluids that change their viscosity under impact, consuming a substantial part of the impact energy [14,21,22,23]. However, these solutions cannot currently be mass-produced, and researchers’ interest is equally focused on high-performance fibres, usually organised in fabrics [24,25,26,27,28]. For stab protection, polymeric fibres are used [29], as well as carbon fibres [30] and metallic fibres [31], sometimes in bold combinations [32], with natural [33] but effective fibres. Three-dimensional architectures of fabrics have also been proposed [34]. A recently accepted solution, due to its successful experimental results, has been the use of fabrics coated with abrasive particle composites, which can increase the energy absorbed by friction [35,36]. Ergo, the objective of this paper is to present the behaviour of a material made of aramid fabrics, coated with a composite of abrasive particles.

In a recent study [37], different aramid laminate architectures were tested for evaluating their capability of resistance against stabbing with knife type S1, the same threat used in this study. Tests were performed on a striking energy range of 0–5 J because the samples have only three layers of woven fabrics or/and prepeg (fabrics with thermoplastic coating). The results allow for pointing out the influence of combining the two types of fabrics and their sequence in the total arrangement. Samples are 160 mm × 160 mm. The weaving of the tested fabrics was both the same type of plane, with one not treated and the other one coated with thermoplastic. The authors used gelatine as a backing material, not sponge or foam as in [NIJ], but this method helped visualise knife penetration. These types of tests helped researchers to rank the designed arrangement solution. Using these samples, the failure mechanisms were explained as a function of the striking energy level and the architecture of the panels.

The particularly high limit values of aramid fibres, tensile [38] and shear [39], especially dynamic loading, make them a main component of body armours [40,41,42,43] when facing impact [44], ballistic [45,46,47,48], stabbing, or spiking threats [49].

The threat posed by a knife depends, among other things, on its sharpness, style, handle and blade design, angle of attack, the physical condition of the attacker, and the skill of the attacker. Because these parameters can vary greatly from situation to situation, weapons that will stop a standard test blade may not stop other knife designs under similar conditions or the same knife design if other attack parameters are altered [1,50].

A classification of tests carried out by the researchers [51,52,53] for new design solutions in the field of body protection may be seen as follows:

- Tests on usually smaller samples, which point out and rank the stabbing, puncture, or ballistic responses of the panels, without too closely mimicking the conditions of real attacks, such as tests on drop-test machines, where impact energy, velocity, and time can be accurately measured but do not realistically mimic the grip or support (materials are not close to body response) of panels;
- Tests according to the accepted standards for assessing stab resistance under conditions closer to reality;
- Tests required by the beneficiaries with different weapons or projectiles.

The aim of this research is to characterise panels, in terms of stab resistance, using a material that has recently been released on the protective equipment market and in a configuration (number of layers) that could be used for actual body armour. The novelty of this report is that includes experimental tests on two impact test installations and investigations into the failure mechanisms of this material. Based on the experimental results, the authors established the influence of panel thickness on the impact characteristics with a knife weapon for two types of tests (on smaller panels on a drop-test machine and on larger panels on an installation complying with the NIJ Standard 0115.00 Stab Resistance of Body Armor [54] and NIJ Standard 0115.01. Stab Resistance of Personal Body Armor (Draft for Public Comment) [55]). The parameters of interest were measured and discussed for assessing the protection quality (maximum force, absorbed energy, time of the stab process, and depth of penetration into the support material).

## 2. Materials and Methods

### 2.1. Materials

The material used in this research paper is supplied by Teijin Limited (Teijin Aramid BV, Arnhem, The Netherlands) [56,57], which developed Twaron^®^ Microflex and Twaron^®^ SRM. The first is specially designed for correctional officers and is made from Twaron^®^ microfilament yarn (550 dtex—type 2040), recommended for knife and spike attacks. The second is also a unique material offering better protec tion against a wide range of weapons. Twaron^®^ SRM incorporates Twaron^®^ CT microfilament fabric with a composite coating (a polymeric matrix and embedded silicon carbide abrasive particles). These fabrics could be also used for correctional officers’ protection.

**Figure 1:**
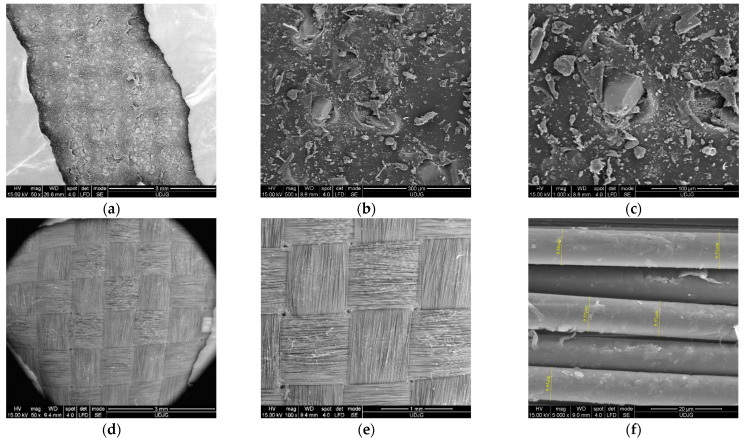
SEM images of the fabric SRM509. (**a**–**c**) The front of the fabric: (**a**) front view (the abrasive coating), ×50, (**b**) abrasive particle (×500), (**c**) abrasive particle (×1000). (**d**–**e**) The back side of the fabric: (**d**) fabric back, ×50, (**e**) detail of the 1/1 woven fabric back, ×100, (**f**) measured diameters of the aramid fibre, between 9.05 μm and 9.9 μm, ×5000.

In this paper, the used Twaron^®^ SRM fabric was coded as SRM509 in order to shorten the supplier designation Twaron^®^ Fabric Style SRM 509, which has the following characteristics: coated fabric surface density of 430 g/m^2^, linear density (dtexnom) of 9300 f1000, abrasive particle surface density of 127 g/m^2^, and fabric roll width of 158 cm. Due to this particular coating, the material SRM509 is flexible as a nearly uncoated fabric. The material has a density that is only a quarter of that of solutions on the market (e.g., steel) [57]. Figure 1 presents SEM images, at increasing magnifications, from the front and back faces of the fabric SRM509. The first line of the images (a–c) is from the front of the fabrics, i.e., the face that be attacked by the knife, and the second line (d–f) presents images from the opposite side of the fabric.

**Figure 2:**
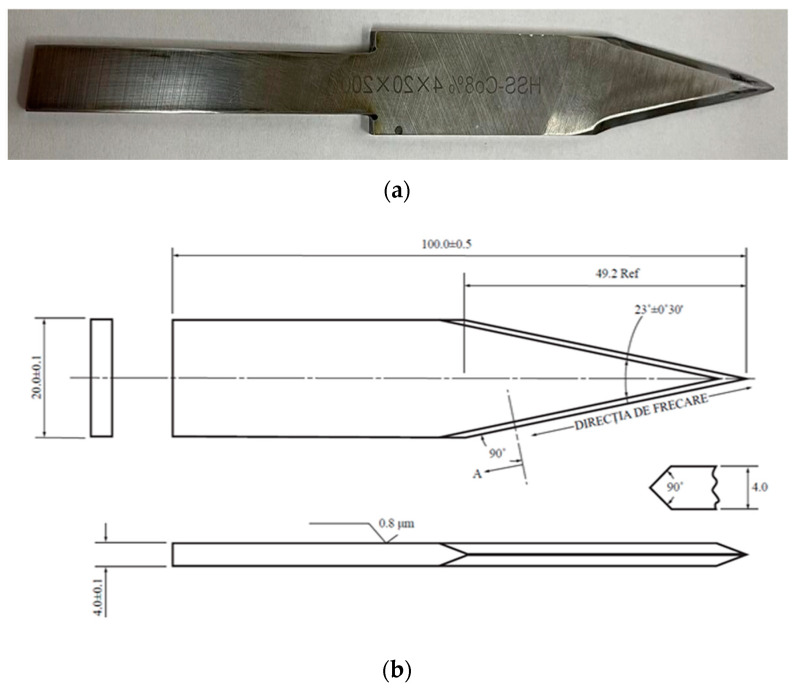
The knife S1 used in stabbing test: (**a**) manufactured and (**b**) the technical drawing of the knife (dimensions in mm from [54]).

White weapons for stabbing and spiking testing are recommended to be made of BS 4659 BO1 steel [58], hardened and tempered to a hardness of 52–55 HRC for the knife blade, as demonstrated in [55]. Typical applications include the following: tools, punches, punching tools, active components of dies, and stamps. For this study, the authors selected the trademark steel B01 HSS-Co 8%, 52…55 HRC, imported by Proma Machinery SRL Romania, the producer being Fervi, Italy. Table 1 gives the chemical composition of this steel grade. The initial dimensions of the plate were 20 mm × 4 mm × 200 mm. The steel, as treated, offers sufficient characteristics, including good and constant surface hardness after tempering, good wear characteristics, dimensional stability on hardening, and machinability. It is equivalent to AISI 01, GB 9CrWMn, JIS SKS3, ASTM A681, DIN 17350, BS ISO 4957:2018 [58]. The knife geometry was that as shown in [55] (Figure 2), a geometry characteristic for two-blade commando weapons or larger kitchen knives.

### 2.2. Methods

For this study, two types of tests were conducted: tests that help rank materials or panels on the Instron^®^ CEAST 9350 drop-tower impact system from National Institute for Aero-space Research Bucharest (INCAS) by using smaller samples and particular fixing, andests that are closer to reality on the facility from Center for Research and Innovation for CBRN Defense and Ecology (CRICBRNDE), which emphasises the importance of testing for bladed weapon attacking resistant materials (samples with actual dimensions and backing materials that resemble the human body).

**Figure 3:**
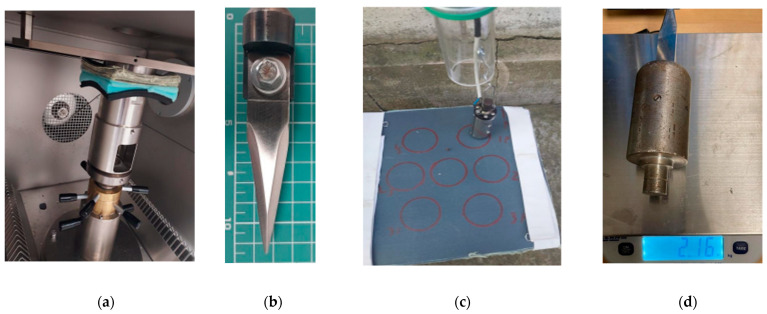
(**a**) Fixture of the test package (panel, witness paper, backing package) on Instron^®^ CEAST 9350 drop-tower impact system, (**b**) knife mounted in the device used on drop-tests, (**c**) sample and the end of the drop-tube and the device for mounting the knife on the facility from Center for Research and Innovation for CBRN Defence and Ecology (CRICBRNDE), and (**d**) the device with the fixed knife and the mass of the dropping device (here in kg).

Figure 3a presents the fixing of the panel and backing support in the Instron^®^ CEAST 9350 frame. The package is pressed with 0.3 MPa between two steel rings. This fixture is very different from that those produced in the facility from the Center for Research and Innovation for CBRN Defense and Ecology (CRICBRNDE), where samples are fixed with elastic bands (of white colour in Figure 3c, and the package is on a ballistic plasteline bed).

The same auxiliary materials were used for both sets of tests on the Instron^®^ CEAST 9340 drop-test machine and on the facility from CRICBRNDE.

Body armour used by personnel in law enforcement organisations must face frequent and very different threats; thus, researchers are focused on new materials and new design solutions [59]. Body armours could be classified by taking into account their flexibility, wearing styles, wearing situations, protection level, and the threat (ballistic, stabbing, puncture, blast, etc.). Even if there are very bold design solutions reported in the literature [1,16], the authors think that stratified panels made of fabrics with high-performance fibres, either with adequate coatings or not, will be preferred because of several advantages like flexibility, lower areal density, and higher productivity.

The panels tested by the authors consist of several layers of fabric SRM509, clamped in the corner with a cross sew of aramid yarns.

The backing package consists of four layers of neoprene sponge, a single layer of closed-cell polyethylene foam, and two layers of rubber. In this order, they are arranged on the witness paper. Each test has its package of tested anti-stab panels (with different number of layers), a PolyArt paper between the tested panel and the backing support. Table 2 presents the characteristics of these materials, close to those recommended in [55].

The same auxiliary materials were used for both sets of tests.

The support (backing) package consists of several layers of the material as follows: the striking surface of four layers of neoprene sponge, a single layer of closed-cell polyethylene foam, and two layers of rubber.

For the backing material, the recommendations from NIJ 0115.00 were taken into account [55], and the supply possibilities and the selected materials for the test backing package are given in Table 2 with their characteristics.

The package of neoprene sponge is recommended in the American NIJ Standard 0115.00 [55] as 4 pieces of 6 mm each (24 mm in total). In the present work, 3 pieces × 8 mm = 24 mm were used to form an equivalent pack.

The test paper is a special paper that does not unravel after testing, and, therefore, the cut length is realistically measured as the cut length in this paper. In the panel and sponge plates, the material relaxation effect screens the actual cut length. The quality of the paper is characterised by the following values: surface density of 140 g/m^2^ and thickness of 0.178 mm. PolyArt paper supplied by Antalis SA Bucharest (Bucuresti, Romania) was used, which was delivered and cut in sheets of 130 mm × 130 mm and 400 mm × 400 mm, corresponding to the sizes of the tested samples. PolyArt is a synthetic “paper” made of HDPE (high density polyethylene), treated on both sides, durable, and water and mechanical resistant. It can be printed offset or by thermal transfer. It is resistant to wet conditions, guaranteed for contact with food, and complies with toy protection standards. It has the advantage of being water and grease resistant. Tien [59] used witness paper sheets between each sponge plate under an anti-stab panel. In this work, only one marker paper was used, placed between the back of the panel and the first 8 mm sponge plate. The standard NIJ 0115.00 [55] also recommends a single sheet of PolyArt paper placed between the sample and the backing material for measuring knife penetration.

**Figure 4:**
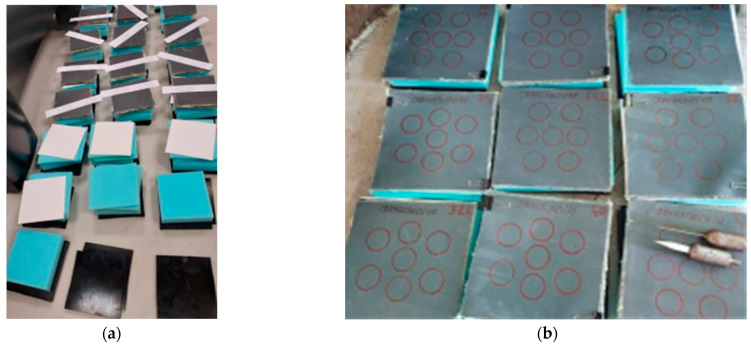
Packages to be tested for this study: (**a**) with Instron^®^ CEAST 9350 drop-tower and (**b**) with CCIACBRNE facility (on the down-right package, there are two weapons (knife and spike), mounted in their device).

Figure 4a presents the smaller set to be tested and circles drawn in Figure 4b on larger samples have diameters and a distance between the central point of two centres as those recommended in NIJ 0115.01, 2020 [54].

### 2.3. Data Analysis

**Figure 5:**
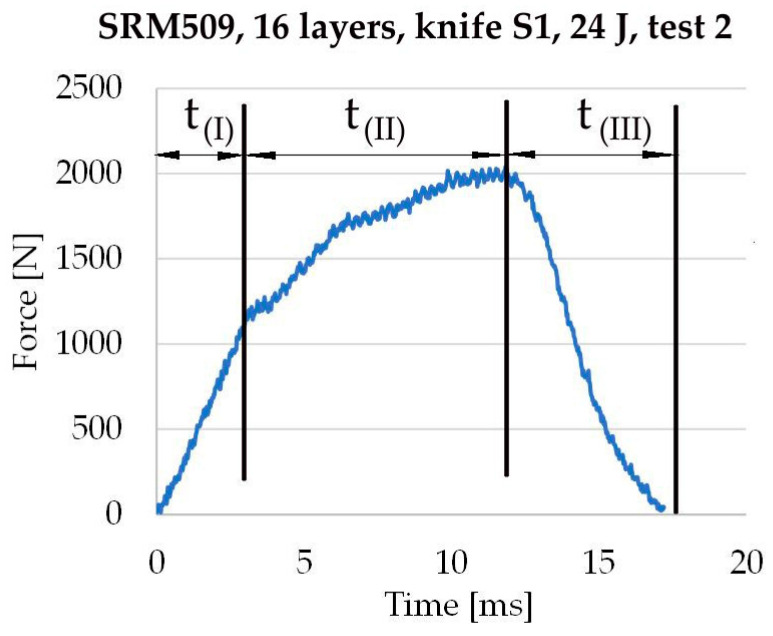
Stabbing stage durations, established on an actual test force–time curve and the notations used in the test campaign on Instron^®^ 9350 drop-tower impact system.

The perforation mechanism of the fibrous material and layered structure differ from woven or knitted fabrics; therefore, the compression value of the fixing structure makes a critical change in the package density under the perforating blade and the area around it. During the stabbing process, the fibre stretches and entangles with the blade. The drag force is the compressive force on the tip of the cutting blade [60]. Analysing the force–time curve (as that presented in Figure 5), for the stabbing process, three stages could be delimited, each being characterised by a time interval and the curve shape.

On the force–time diagram (for tests on Instron^®^ 9350 drop-tower impact system), both for the knife, three stages are clearly distinguished:

- Stage I: The force increases rapidly, apparently after a straight line;
- Stage II: A slope linking the first and last stage, usually with increasing force;
- Stage III: The force is decreasing to 0 (zero); the curve has the shape of a very narrow and high S.

Stage I, characterised by duration t(I): When the knife tip touches and then advances contact with the panel, a compression zone is generated under the tip, evidencing the elastic characteristic of the panel; this interval is short, and the yarns remains intact.

Stage II, characterised by duration t(II): With the movement of the perforator blade, the fibrous structure becomes more compressed, and the fibres become more strained to resist perforation; there is a change in the shape of the curve due to the elasto-plastic components of the strain. Fibre tensioning and bending are caused by the frictional force of the blade surface and friction with other surrounding fibres, both still intact and cut. The movement of fibres during the penetration of the blade is largely responsible for the difference in the perforation behaviour of the fibrous structure. But, as the blade advances, it starts to cut more and more fibres or bunches (yarns) of fibres. This process of shear cut of the fibres has a different smaller slope as that in the first stage, usually being ascendent in time, meaning that the blade had to increase its force to cut more and more layers. The force also includes the component capable of resisting friction between the fibres and the blade and friction among fibres.

Stage III, characterised by duration t(III), is the final stage of the stabbing process. Only lateral zones of the blades (for knife S1) enlarge the cut as the blade advances, and the fibres in direct contact with the blades are cut, set aside, tensioned in traction and/or bending, entangled. Subsequent failures of fibres adjacent to the blades occur in all directions. If the fibre architecture has a high packing density (as for 1/1 fabrics) and the fibres are long and tightly packed, the stabbing resistance will be the highest as most of the fibres under the blade will be cut.

The following points can be identified on the force–time curve:

- The initial point of the stabbing process, when *t*_0_ = 0 and *F*_0_ = 0, both values are considered null but are the last null value of the force in the string of (*t*, *F*) pairs. After this null value, the force is increasing (the force *F* oscillates around zero during the distance travelled until the blade tip hits the panel, so it is the moment of the last zero value of the force before it starts to increase).
- *t_f_* is the moment when the force *F* reaches the first zero value after the blade strike, so *F*(*t_f_*) = 0; thus, the stabbing process ends at this moment.

**Figure 6:**
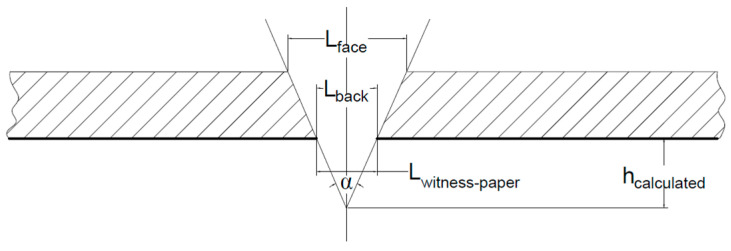
Sketch for calculating the knife penetration height: *L_back_*—cut length on the panel back, in mm, *L_witness paper_* or *L_paper_*—cut length on PolyArt paper, in mm.

In the American standard NIJ 0115.01:2020 [54], the parameter assessing the stab resistance is the depth (or height) of knife penetration under the panel. This is calculated based on Figure 6 with the following relationship derived from the geometry:

(1) $$tg\frac{\alpha }{2}=\frac{\frac{L_{paper}}{2}}{h_{calculated}}\;\to \;h_{calculated}=\frac{L_{paper}}{2}\frac{1}{tg\frac{\alpha }{2}}$$

where *h_calculated_* is the length of the measured cut on the witness paper in mm, and *α* is the angle characterising the tip of the knife S1, as in NIJ 0115.01:2020 [54].

Tests performed on the facility will be characterised by geometrical parameters as *h_calculated_*, *L_witness-paper_*, *L_face_*, and *L_back_*. This facility, although not instrumented, meets the NIJ Stantard 0115.01 recommendations for energy levels, clamping systems, and sample sizes.

**Figure 7:**
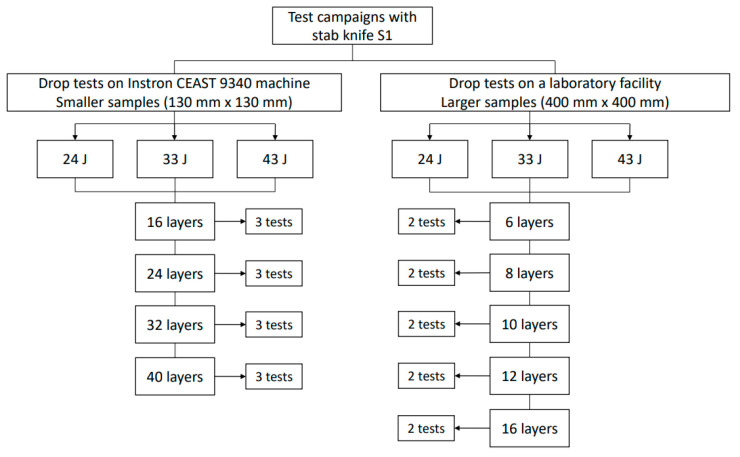
Test campaigns for the stab tests.

The two different test campaign are given in Figure 7.

The authors started with the campaign involving the small samples tested on the Instron 9340 machine. The tests on the facility from the Center for Research and Innovation for CBRN Defense and Ecology (CRICBRNDE) began with the panel with 16 layers of fabric SRM509, and we noticed from the first test that the same knife, type S1, did not cut the last layers. So, testing thicker panels, as in the previous campaign for smaller samples, was not of interest because they will have the same result: the blade does not cut all the layers in the panel. This will not help us to see how these panels behave when they have a smaller number of layers. Also, it was of interest to determine whether the number of layers exceeded the “red” values of 7 mm for the penetration height. In NIJ 0115.01 [54], the section, “Armor Performance Requirements”, provides recommendations. Each tested sample “shall experience no penetration greater than” the following threats.

- Commercial threats:
  ○ 7 mm at primary energy levels E1 (with values of 24 J, 33 J, and 43 J, respectively) for fair hits at incidence angles of 0° and 45°;
  ○ 20 mm at overtest energy levels E2 for fair hits at angles of incidence of 0°.
- Improvised threats:
  ○ 0 mm at E1 for fair hits at angles of incidence of 0° and 45°;
  ○ 20 mm at E2 for fair hits at angles of incidence of 0°.

This study only focused on the primary energy levels E1 (meaning tests with a strike energy of 24 J, 33 J and 43 J, respectively, and at incidence angles of 0°) for both test campaigns, and a roll of fabric Twaron^®^ SRM509 was used, with a total surface area of the fabric of 150 m^2^.

## 3. Discussion of the Test Results

### 3.1. Data from Tests on Instron CEAST 9340 Machine

**Figure 8:**
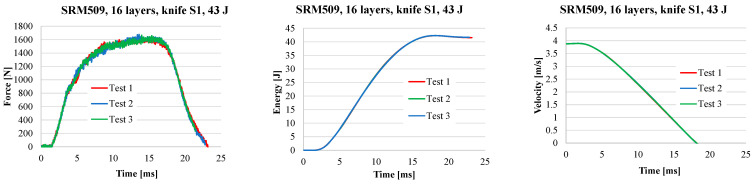
Force–time, energy–time, and velocity–time curves for the panel with 16 layers of SRM509 for three tests with the same input parameters (knife S1, 43 J, and panels with 16 layers).

Figure 8 presents typical force–time curves for different number of layers in a panel, namely, 16, 24, 32, and 40. A typical curve in this study is one of the three curves determined under the same conditions (number of layers, strike energy, and velocity), with the difference among these three curves being small.

**Figure 9:**
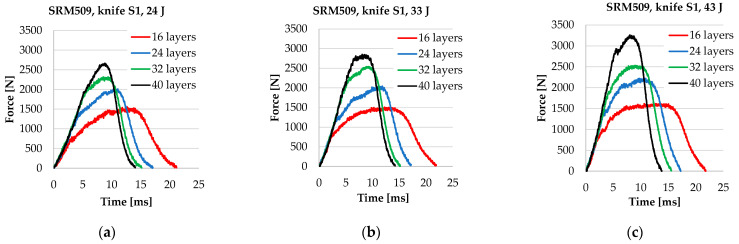
Curves force–time for tests with different numbers of layers for: (**a**) 24 J, (**b**) 33 J, and (**c**) 43 J.

From the force–time curves (Figure 9), the following observations could be formulated:

- The values for *F_max_* increase with the number of layers for the same striking energy;
- The first part of the curves is almost linear and overlap;
- Time of the stabbing process is decreasing when the number of layers increases;
- The stabbing time is less sensitive to the striking energy;
- The plateau characterising the second stage of the stabbing process becomes narrower when the number of layers increases because, for the thinner panels (16 layers), the failure of the layers unravels one by one, or at least in a larger time interval, with the panel being more flexible; for thicker panels, as they have a higher non-deflecting behaviour, the knife destroys them quicker.

**Figure 10:**
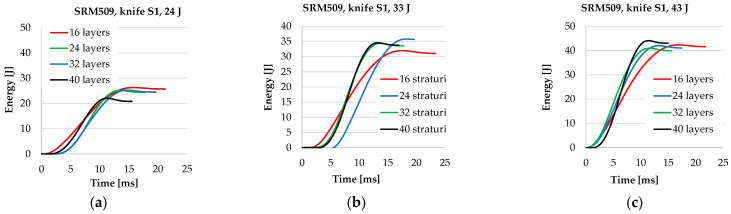
Energy–time curves, depending on the number of layers in a panel and strike energym for (**a**) 24 J, (**b**) 33 J, and (**c**) 43 J.

**Figure 11:**
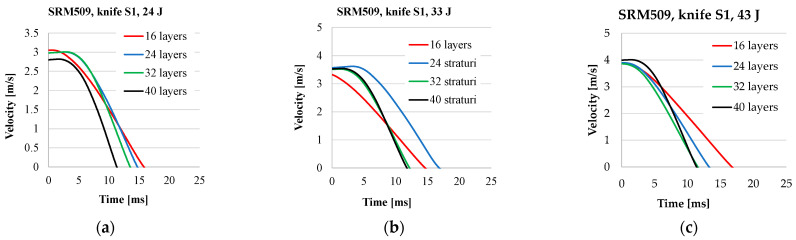
Velocity–time curves, depending on the number of layers in a panel and strike energy, for (**a**) 24 J, (**b**) 33 J, and (**c**) 43 J.

A study published in 2018 [61] shows that forces generated by volunteers in mild, moderate, and severe stabbing tests were, in almost all cases, significantly greater than the forces required for skin penetration. They suggest that the force required in any stabbing process is influenced by the following factors: the radius of the weapon tip, the minimum force required for penetration, the sex of the aggressor, and its hand (dominant or non-dominant). The variability of the factors involved in the stabbing process has also been studied in [62,63,64]. The forces achieved by the volunteers are lower than the forces recorded for the destructing samples using energies recommended in the standards (typically from 24 J to 43 J) [61]. The report published by Nolan et al. [61] gave a maximum stab force of 800 N for a male stabbing with his dominant hand. Analysing the values of maximum force in Figure 10, as measured in this study, on the Instron machine, one may notice much higher values, starting from 1500 N for tests on panel with 16 layers (for all three energy levels) to 3300 N for tests on panels with 40 layers, impacted by an energy of 43 J. In Figure 9, Figure 10 and Figure 11, the initiation of the stabbing process is the same, *t*_0_, the moment when force *F* has its last null value and then increases.

The thinner panel has a shallower slope in the energy–time curves compared to the thicker panels, meaning energy absorption is slower. Also, the time necessary to stop the weapon is longer. The energy–time curves for panels with 32 layers and 40 layers are closer, meaning that under the same conditions, the influence of the number of layers in the thicker panel is diminishing. And the time intervals necessary to absorb all the strike energy are close for these two panels.

The velocity of the knife tip is plotted starting for the time *t*_0_, the initial point of the stabbing process, as defined in subchapter 2.3 Data Analysis. Of course, there is a rebound of the knife and the machine record a negative value of the velocity, but this is not given in Figure 11.

**Figure 12:**
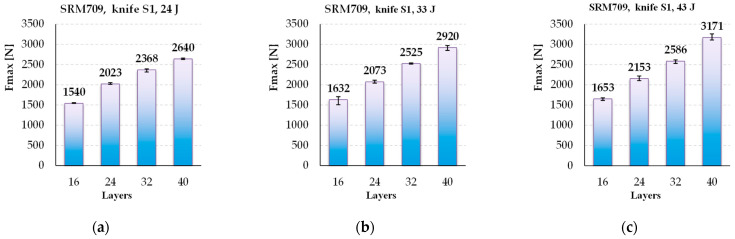
Average values for *F_max_*, depending on the number of layers in a panel, for different strike energy levels: (**a**) 24 J, (**b**) 33 J and (**c**) 43 J.

An instrumented test machine such as Instron^®^ CEAST 9350 drop-tower impact system allow for analysing parameters that changes in very short time periods. Figure 12 presents the average values of *F_max_* (for three tests), and one may notice that this force increases almost linear to the number of layers for all tested energy levels. The following conclusions express percentage, having the value of the same parameter recorded or calculated for 24 J as the basis. This observation is consistent only for the numbers of layers of 16 to 40. From 24 J tests to 43 J tests, *F_max_* increases with only 7.33% for panels with 16 layers and with 20.11% for panels with 40 layers. This means that *F_max_* increases faster for thicker panels.

**Figure 13:**
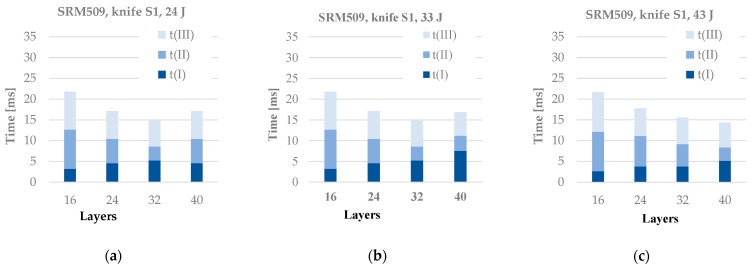
Durations of the stabbing process as the sum of stages identified on test curves (t(I) + t(II) + t(III)) for different strike energy levels: (**a**) 24 J, (**b**) 33 J, and (**c**) 43 J.

Figure 13 presents the duration of the stabbing process, but each value (column) also presents the durations of the stab stages. This parameter is not sensitive to the energy level. For the panels with 16 layers, the values of the stabbing process are close. There is a tendency of reducing the stab time from the thinner panel to the panel with 32 layers, but for the panel with 40 layers, this time increases. All values are in the range of 15–22 ms. Time t(I) is longer than 5 ms only for the panel with 40 layers at an energy level of 33 J. Time t(II) generally decreases with the increase in layer number, and t(III) has small variations, except for the thinner panel.

**Figure 14:**
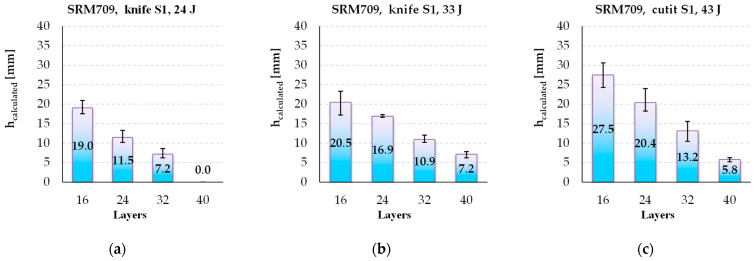
Average values for *h_calculated_*, depending on the number of layers and strike energy, for different strike energy levels: (**a**) 24 J, (**b**) 33 J, and (**c**) 43 J.

The parameter *h_calculated_* is of critical importance when testing the stab behaviour of a panel. Figure 14 shows a clear and almost linear dependence on the number of layers. For the panel with 16 layers, this parameter increases with 44.7% of the energy level of 24 J and 43% and with 83.3% for the panel with 32 layers. At the lowest energy level, 24 J, the panels with 40 layers have no marks of penetration on the back of last layer.

**Figure 15:**
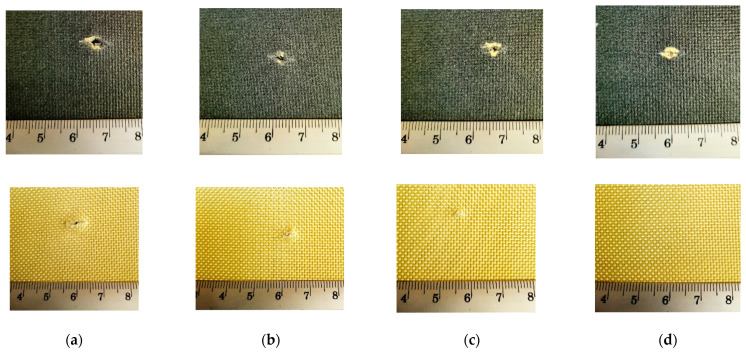
Macro photos of the damaged zone of panels made of SRM509 fabric, with different number of layers, after being tested at energy level of 24 J with knife S1 (**up**—panel front, **down**—panel back): (**a**) 16 layers, (**b**) 24 layers, (**c**) 32 layers, and (**d**) 40 layers.

Analysing Figure 15, which presents a set of photos for panels tested at a strike energy of 24 J, one may easily notice the cut lengths on both faces of each panel (face up and back down). For the panel with 40 layers, the last layer has no mark produced by the knife blade.

**Figure 16:**
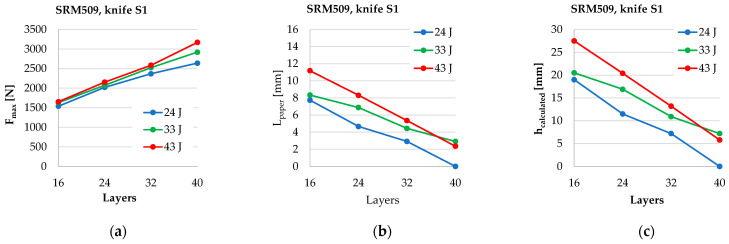
A graphical synthesis of the results obtained on Instron^®^ 9350 drop-tower impact system: (**a**) average values of *F_max_*, (**b**) *L_paper_*, and (**c**) *h_calculated_*.

Figure 16 presents three parameters represented by the average values from the three tests in order to evaluate the influence of the number of layers and the strike energy level. For the same number of layers, *F_max_* has a more differenciate increase for the panels with 40 layers. A greater difference between the values were obtained for an energy level of 43 J. Parameters *L_paper_* and *h_calculated_* have the same evolution. They decrease when the number of layer increases. For the panels with 40 layers, the differences between tests of 33 J and 43 J are small, of 2 mm.

### 3.2. Data from Tests on Facility from the Center for Research and Innovation for CBRN Defense and Ecology (CRICBRNDE)

**Figure 17:**
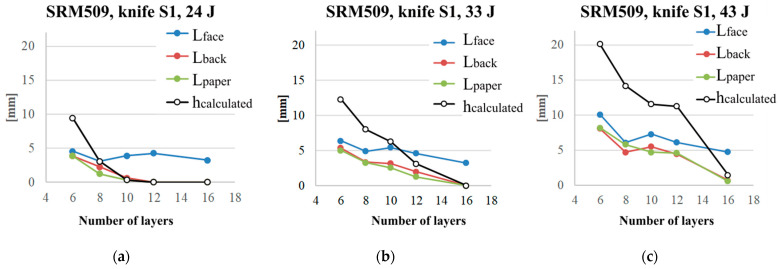
Experimental results (mean values) as a function of the number of layers of SRM509 fabric (tests performed on the CCIACBRNDE facility) for different strike energy: (**a**) 24 J, (**b**) 33 J, and (**c**) 43 J.

The test campaign on larger samples (400 mm × 400 m) and different fixing systems starts with the panel of 16 layers, the authors presumed that thicker ones should be tested. Several repeated tests at 24 J produced no marks on the back of last layer of the panel. Thus, the authors reconsidered this test input and the number of layers and tested the panels with less than 16 layers. Figure 17 presents the length of the cut on the face and back cut length of the panel, and the length from the witness paper and the penetration height were as calculated (the plot values are average of two tests under the same conditions). All geometrical parameters are given on the same plot for an energy level. As the weapon was standard (knife S1 from [41]), the discussion takes into account the penetration height of 7 mm. For an energy level of 24 J, the panels with 8 to 16 layers fulfil the requirement of having less than 7 mm for the calculated height penetration. Only the thinnest tested panel of six layers has this parameter as greater than the standard value.

For the energy level of 33 J, the panels with 10 layers have an average value of *h_calculated_* = 7.47 mm, and the panel with 8 layers has *h_calculated_* = 7.91 mm. For these two cases, supplementary tests should be carried out. The test campaign that will follow these tests will prepare samples of the size as recommended in the standards (500 mm × 500 mm for preliminary tests and then test on prototype) [54]. At 43 J, only the panel with 16 layers has less 7 mm of the penetration height, with very high difference between the panels with 12 layers and 16 layers. More tests will smoothen the graph, but these tests, as presented, are useful in establishing the realistic ranges for parameters, such as the number of layers.

**Figure 18:**
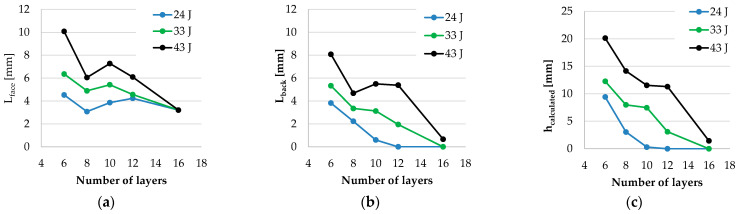
Experimental results (mean values) as a function of the number of layers of SRM 509 fabric after being stabbed with knife S1: (**a**) front length, (**b**) back length, and (**c**) *h_calculated_*.

Figure 18 presents, in separated plots, the geometrical features of the cut: *L_face_, L_back_*, and *h_calculated_*. This last dimension is calculated by taking into account the cut length measured on the witness paper. The lengths measured on the panels have the same trends, with *L_face_* greater than *L_back_*, especially for thicker panels. Analysing Figure 18c, one may suggest testing the intermediate values for the layers. For instance, a panel of seven layers could have *h_calculated_* < 7 mm, but this has to be tested. The same logical step for panels of 14 layers and 15 layers at an energy level of 43 J.

**Figure 19:**
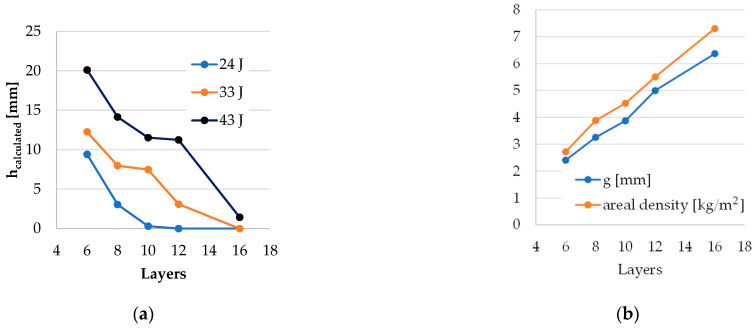
Evolution of several characteristics of panels tested on facility from Center for Research and Innovation for CBRN Defense and Ecology (CRICBRNDE): (**a**) *h_calculated_*, depending on the number of layers and strike energy, and (**b**) measured values for panel thickness and areal density as a function.

The conclusion for this campaign will be formulated based on Figure 19. The discussion is only for knife S1 [41]. For an energy level of 24 J, samples of 8 to 16 layers have height penetrations of less than 7 mm. For an energy level of 33 J, the panel with 10 layers has *h_calculated_* = 7.47 mm. Numerous more tests for this number of layers should be conducted in order to establish more reliable statistics.

For an energy level of 43 J, only the panel with 16 layers fulfils the standard requirement for height penetration. Analysing the tendency of this parameter, one may suppose that a panel of 14 or 15 layers could also have this parameter when under 7 mm. The thickness and areal density of the panels obtained with the layers of the SRM509 fabric have promising values. It is worth testing hybrid panels, meaning combinations with other high-performance fabrics, in order to further reduce the areal density for a specific threat level. Areal density is calculated by dividing the mass of the panel with its area. All data in Figure 19b are obtained by measuring the panel mass and thickness, not by multiplying the thickness of a layer or using the value of density in the fabric datasheet.

### 3.3. Stabbing Failure Mechanisms

The kinetic energy absorbed by the panel, *E_absorbed_* is defined by the following six different components: *E_S_*—yarns shear energy, *E_D_*—deformation energy of all other yarns, *E_T_*—the energy up to tensile rupture of directly affected yarns, *E_F_*—energy required to overcome the friction between the fabric yarns/layers, *E_J_*—energy required to overcome friction between the blade and the yarns, and *E_M_*—energy required to displace the fabric during impact. So, it can be written as follows [1]:

(2) $$E_{absorbed}=E_{S}+E_{D}+E_{T}+E_{F}+E_{J}+E_{M}\;\;$$

At the time after impact, the puncture force value will increase until full penetration and then gradually decrease. To increase the fabric absorption energy, it is expected to evolve each component of the fabric absorption energy, increasing the friction between the blade and the fabric yarns.

**Figure 20:**
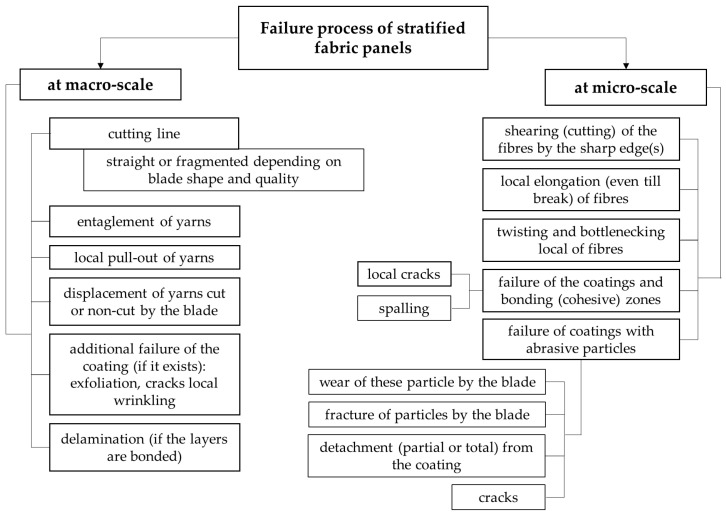
Stabbing failure processes taking into account the abrasive coating.

Based on the studied literature and the analysis of macro and SEM photographs taken on the tested samples, Figure 20 shows synthetically the failure processes occurring when stabbed with a cutting blade-type weapon (symmetrical blade, type S1 according to [54]).

Analysis of the S1 knife stab failure mechanisms at micro-scale level, for the panels made of fabric SRM509, is based on electron scanning microscopy.

**Figure 21:**
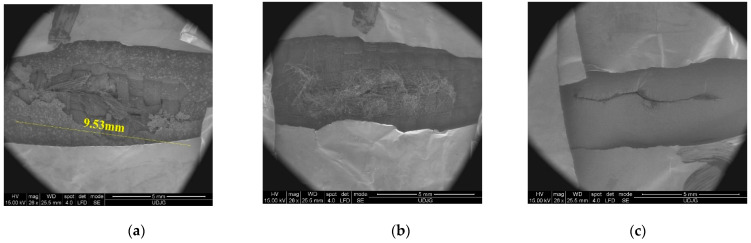
SEM images of the panel with 16 layers of fabric SRM509 (striking energy 24 J): (**a**) 1st layer, front, (**b**) 16th layer, back, and (**c**) witness paper.

Figure 21 shows, at the same scale, the cuts made of the knife S1 in the first layer (a) and the last layer (b) and the cut on the witness paper (c). All cuts are not straight lines, this being caused by the yarns’ architecture (simple woven or 1/1).

Squared parts were cut from the small samples, having at most a side length of 4 mm, from each layer of the tested panels, keeping the cut in the centre of the area. The faces of these parts were coated with a very thin golden film in order to avoid particular aspects when investigating polymeric materials.

For this study, different magnifications were used. For instance, a small magnification (×25), as shown in Figure 21, reveals (a) the brittle nature of the coating, even if at a macro scale, in which the fabric is just a little less flexible as the simple aramid fabric of the same area density. Statistically, the fact remains that yarns perpendicular to the blade width are cut and that those parallel to the width are only entangled and laterally pushed. Moreover, (b) the different aspects of the back of the last layer (fibres are very spread, straggly, cut but, obviously, after being bent, stretched) and (c) even the paper cut are not linear, but the ends of the cut are very clear, and no lateral cracks are noticed.

**Figure 22:**
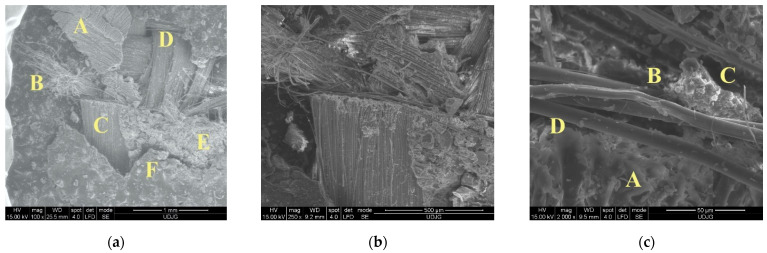
SEM images of layer 1—front of the 16-layer panel of SRM509 after S1 stabbing with 24 J.

Figure 22 presents the failures on the first layer of the panel with 16 layers. Each letter on the image is explained as follows:

(a) Image of the left end of the cut: A—a rotated fragment of the abrasive coating because of the blade action, in which one may notice the shape of the fabric yarns; B—a bunch of fibre, cut at different lengths, in which their positions suggest a small rebound of the knife; C—a yarn perpendicular to the blade width, with fibres cut at the same length (like a guillotine); D—the yarn next to that in C is also cut, but the fibres are a little disordered, possibly because of the friction with the blade and because the blade tip displaces the yarn in its direction of movement: E—small fragments from the coating, with fibres still bonded to them, in which they could increase friction when the blade continues its displacement; F—fracture line of the abrasive coating;
(b) A detail (×250) of yarn C from the previous image;
(c) Failures: A—micro-fragments of the coating; B—a locally stretched (strangled) fibre because it was tensioned in traction, and its position is along the blade surface; C—another fragment from the coating; D—a fibre without visible damage but with very small fragments from fabric coating.

**Figure 23:**
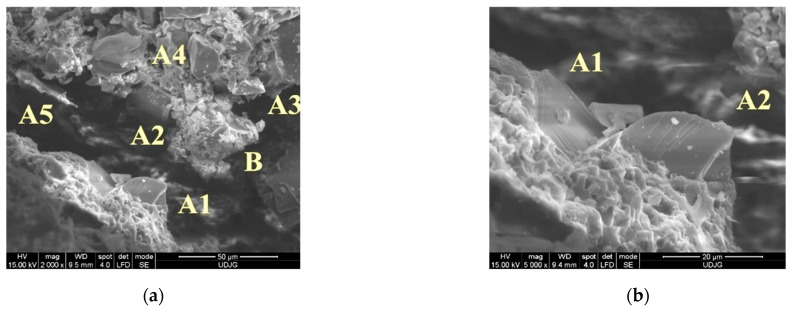
Details of abrasive particles sheared off from S1 knife cut, with an energy of 24 J, on layer 1—front of 16-layer panel of SRM 509.

Figure 23 shows the following details of abrasive particles at the cut edge, partially detached from their matrix, when the knife cuts with an energy of 24 J on layer 1 (front view) of the panel with 16 layers of fabric SRM 509: (a) abrasive particles denoted A1, A2, A3, A4, and A5 could, with a high probability, having been in contact with the knife body, generate an abrasive friction that consumes the kinetic energy of the weapon and B, the surface of the fractured matrix that could, too, increase friction; (b) at a magnification of ×5000, two hard particles can be seen, right at the edge of the cut, so they most likely interacted with the knife body.

**Figure 24:**
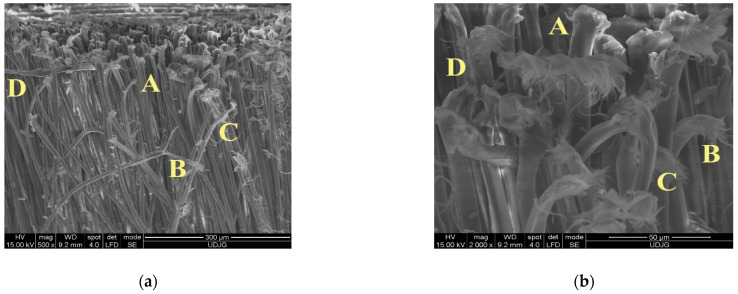
Details of shear-broken fibres from the S1 knife cut, with an energy of 24 J, on layer 1—back of the 16-layer panel of SRM 509.

Figure 24 presents the details of a cut yarn on the back of the layer 1 from the panel with 16 layers as follows:

(a) A—typically cut aramid fibres (the cut end of the fibre is like a nail head), B—a fibre that was broken after fibrillation meaning that it was also stretched and bent before breakage, C—fibre twisted with a thinner local section, and D—very rare failure for an aramid fibre consisting of flattening, meaning the main load was of a compressive nature;
(b) Detail of the cut fibres: A—a neat sheared fibre, B—the end of this fibre is sheared but also bent, probably after being cut because of the friction with the steel body, C—fibre broken by stretching and shearing, with throttled areas and distinct fibrils indicating differences in the degree of crystallinity and/or micro defects in the fibrils, and D—sheared but blade driven fibre with a flattened area, revealing that the cutting edge attacks fibrils one by one but in a very short time.

**Figure 25:**
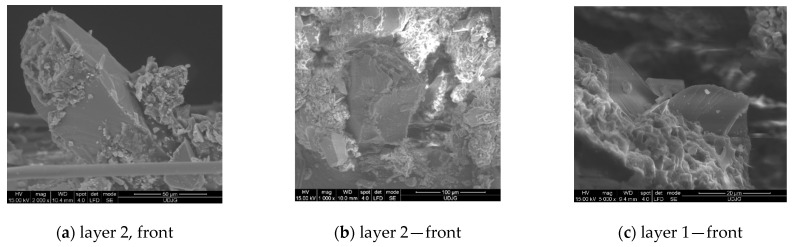
SEM details of abrasive particles broken by knife strike at an energy of 24 J on the panel with 16 layers of fabric SRM 509.

Figure 25 shows details with hard particles located on the cut edge: the observed particles have different sizes and shapes, but with sharp edges, which can scratch the knife blade, consuming more of the impact energy through dry friction, this component of energy dissipation is missing when using uncoated fabrics. Additional details of Figure 25 are as follows: (a) the broken crag of the particle and the light-coloured fracture lines suggests an interaction with the knife blade, meaning a small quantity of the blade energy was consumed for fracture the particle, (b) another particle has a broken corner, still fixed in the matrix, and (c) for sharp edges of two particles on the cut edge, there is a high probability that these edges scratched the knife body.

**Figure 26:**
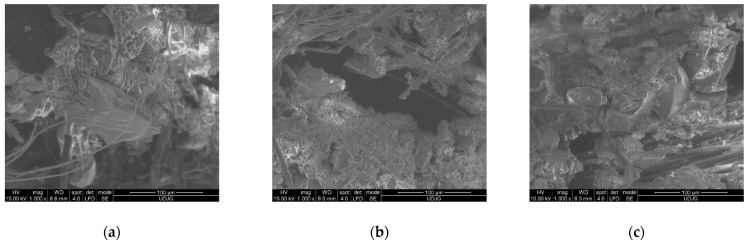
Details of the cut on the layer 16 of the panel with 16 layers (front view).

Figure 26 shows the following details of abrasive particles on the cut edges from the layer 16 from a panel of 16 layers at an energy of 24 J; it is worth noticing the high degree of disorder of the fibres:

(a) For the hard particle, partially detached from the matrix as its upper face still have traces of matrix it was embedded in, visible fibres are locally stretched, and their sharp ends suggest break by traction because they were tensioned before being cutting, when the last layers were pushed in the direction of knife movement;
(b) Left end of the cut;
(c) At a magnification of ×1000, with two hard particles at the right end of the cut, the bigger particle has marks of abrasion and a visible fracture line.

## 4. Conclusions

The experimental results from two test campaigns for evaluating the layered panels with fabric SRM509 emphasise the importance of sample size and fixing. The first campaign on smaller samples is useful when comparing two or more materials under the same test conditions. The second campaign is relevant as the sample size and its fixing are close to standard requirements to be met before producing the prototype. With only 100 linear meters of fabrics, the authors established the influence of the number of layers on the maximum force acting during stabbing, on duration on stabbing process, and on a main parameter of interest, the penetration height. The higher the number of layers, the better the stab resistance performance.

The maximum force duration in the 40-layer test is close to 10 ms, whereas in the 32-layer tests, the maximum force duration is 7 ms (milliseconds).

The variation in the *F_max_* force is not very large from one layer to another. From 24 J to 33 J, the energy jump is 37.5% of the initial value, while the jump for force is a 5.9% jump from the maximum force recorded at 24 J.

The pulling and rubbing force of the yarns limited the slippage of the wires, which caused more yarns around the push to dissipate energy.

The panels with layers of fabric SRM 509 have an almost linear, increasing dependence of *F_max_* on the number of layers in the tests performed at the same energy level. At a higher number of layers, *F_max_* is obtained at the highest energy level.

The influence of the number of layers on *F_max_* is greater than the influence of strike energy.

Scanning electron microscopy (SEM) was selected to analyse the failure mechanisms at a micro scale. We used low and high magnifications for explaining how yarns, fibres, and the particular abrasive coating fail.

A very important conclusion of this study regarding the stabbing performance of fabric Twaron^®^ SRM 509 is that a research study could start on small size samples, with an accurately instrumented machine, in order to establish the influence of significant factors of stab resistance, as the samples are lower in costs and time consuming, but this study continued to examine larger size samples and focused on using the data for the prototype, and repeatability was notably high. These are the requirements to be fulfilled for body armours, products that must face high-risk events.

## Figures and Tables

**Table 1:** Chemical composition of BO1 BS 4659 steel grade [58].

Element	C	Mn	Si	W	V	Cr
Wt%	0.85–1.0	1.10–1.35	0.40	0.40–0.60	Max 0.25	0.40–0.60

**Table 2:** Characteristics of the components for the backing package.

Component	Characteristics	Supplier from Romania
Sponge plate	25 kg/m^3^, type HR, pressure strength 2000 kPa, 8 mm thickness	Intex Conect SRL Giurgiu
Soft polymeric foam	35 kg/m^3^ density, type HR, pressure strength 2500 kPa, 30 mm	Intex Conect SRL, Giurgiu, Romania
SBR rubber technical board—general purpose rubber, without textile insert, working temperature: −30 °C to +70 °C	6 mm thickness, smooth, black, 1400 mm width, hardness 65 ± 5° Shore A, 6 mm thickness	SC Arte Rubber Distribution SRL, Târgu Jiu, Romania

## Data Availability

Data are contained within this article.
